# The Influence of Fear During Pregnancy, Labour and Delivery on Birth Outcome Among Post-Delivery Women: A Case Control Study in Zanzibar

**DOI:** 10.24248/eahrj.v6i2.693

**Published:** 2022-11-30

**Authors:** Mwanaali H. Ali, Saada A. Seif, Stephen M. Kibusi

**Affiliations:** a Department of Clinical Nursing, The University of Dodoma-Tanzania; b Department of Nursing Management and Education, The University of Dodoma-Tanzania; c Department of Public Health, The University of Dodoma-Tanzania

## Abstract

**Background:**

Assessing the influence of fear during pregnancy, labour, and delivery on birth outcomes among women is very important. Normally, women experience happiness during pregnancy, but some may develop fear which may cause maternal and neonatal complications. The aim of this study was to determine the influence of fear during pregnancy, labour and delivery on birth outcome among post-delivery women in Zanzibar.

**Methodology:**

This was a matched case-control study involving 204 post-delivery women who were randomly selected from 4 hospitals in Zanzibar. Cases (n=68) were those who experienced a negative birth outcome, whether maternal, fetal, or both. The control group (n=136) had normal birth outcomes. A self-administered questionnaire was used to collect data and was analyzed using SPSS whereby percentages, chi-square test, and odds ratio results were reported.

**Results:**

Among cases, 27(39.7%) had high level of fear during pregnancy compared to the control group, 75(40.4%). During labour, 29(42.6%) of cases had high level of fear, and in control, 55(42.4%). And during delivery 35(51.4%) of cases had highest level of fear, while only 47(34.5%) of control had high level of fear. The chi-square test showed only fear during delivery was significantly associated with undesirable birth outcomes. Women who experienced a high level of fear during delivery were 2 times more likely to have undesirable birth outcomes (AOR=1.941, *p=.051*) after adjusting for other variables.

**Conclusion:**

This study established that most women experience high level of fear during pregnancy, labour and delivery. A high level of fear during delivery is associated with having negative birth outcomes, but not during pregnancy and labour. The findings are of clinical importance as they highlight the need to integrate a universal screening intervention into antenatal care services for early management.

## BACKGROUND

Having a new life developing inside a woman's body during pregnancy creates a new state of emotions. Pregnancy has been termed as an emotional crisis^1,2^ since it is associated with many different physical and social changes.^3^ Normally, women experience happiness, satisfaction, and self-fulfilment during pregnancy, but may also develop pregnancy-related anxiety which includes fears and worries about, but not limited to, the health and survival of the unborn child, having an abnormal baby, the delivery process, developing medical problems during pregnancy, and the ability to parent and care for the infant following birth.^4^

Pregnancy-related anxiety has attracted considerable research attention and fear is one of its dimensions.^5^ The high maternal and neonatal morbidity and mortality rates in sub-Saharan African countries, poverty, and shortage of resources in hospital settings may lead to a high prevalence of fear related to pregnancy and its effects may be more prominent among post-delivery women. Tanzania has a maternal mortality ratio of 556/100,000 live births ^6^ while Zanzibar has a ratio of 276/100,000 live births ^7^, a fertility rate of 5.1 children ^8^, and 65,688 women of expectant reproductive age.^6^ However, not much is known in many sub-Saharan African countries, Tanzania included, about the fear in pregnancy and its influence on birth outcome.

The worldwide prevalence of pregnancy-related fear of childbirth has been estimated at around 14%. In developed and developing countries, the prevalence of fear is about 10% and 25%, respectively. Pregnancy-related fear is estimated to be around 23% in Alberta, Canada, 15.6% in Germany, and 49% in Pakistan. ^9^

Studies have shown a correlation between fear and undesirable birth outcomes. Fear can end up with fetal distress, premature delivery, and the possibility of a ruptured uterus.^10^ Fear has also been linked to preterm labour, low birth weight, Caesarean Section (C/S) and instrumental deliveries^11^ as well as prolonged labour, post-partum haemorrhage, birth asphyxia and even death.^12^ Fear in early pregnancy may result in fetal loss, and fear in the second and third trimesters leads to a decrease in birth weight.^13^ However, there is not enough information to support the correlation between fear during pregnancy, labour and delivery and birth outcome in low-income countries.

In Tanzania, there is a paucity of information about pregnancy-related anxiety. The only qualitative study conducted in Mwanza-Tanzania showed that women acknowledged experiencing a state of worry and concern during pregnancy, often causing physical symptoms and disrupting their personal sense of peace.^4^ Another is a quantitative study also conducted in Mwanza which showed that about 25% of the study participants scored higher in pregnancy-related anxiety scale and it was predicted by the perceived stress, active depression and number of people living in the home.^14^ There is still a need to explore more about the pregnancy-related anxiety in Tanzania and how much it is associated with undesirable birth outcomes. Therefore, this study intended to assess the influence of fear during pregnancy, labour, and during delivery on maternal and neonatal birth outcome.

## METHODOLOGY

### Study Design and Setting

The study design was a matched case-control. This was a hospital-based study conducted in postnatal wards in Unguja, Zanzibar-Tanzania in national and district hospitals. Unguja has one national referral hospital (Mnazi Mmoja hospital) and two district hospitals (Kivunge and Makunduchi hospitals), all of which offer birth delivery services.

### Study Population, Sample Size, and Sampling

The study involved all post-delivery women admitted and had delivery at the health facilities. Participants in the case and control groups were matched based on age and parity. Cases and controls were defined according to the status of birth outcome.

***Cases definition:*** Having undesirable/negative birth outcome for either mother or infant or both. The undesirable birth outcomes considered in this study were birth asphyxia, caesarean section, fetal distress, instrumental delivery, intrauterine fetal demise, low birth weight, postpartum haemorrhage, premature delivery, prolonged labour, ruptured uterus and severe pre-eclampsia.

***Controls definition:*** are defined as having a normal birth outcome for both the mother and the infant.

***The exposure*** status, which was determined retrospectively, was having a high level of fear during pregnancy, labour, and delivery.

***Non-exposure*** status was having a low level of fear during pregnancy, labour, and delivery.

The study included all who agreed to participate and excluded all known cases of diabetes, renal, and heart diseases among both cases and controls.

The sample size was estimated with a hypothesis of two-sided equality.^15^ The prevalence of fear among women with undesirable birth outcomes of 30% in developing countries was used.^13^



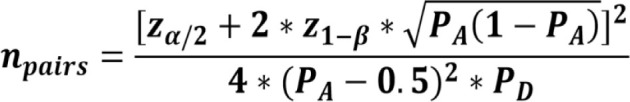



### Variation Notation

n pairs= Sample size pair for case control study

α = Probability of margin error of cases of 5%

β = Probability of margin error of control 5%

P_A_ = Population proportion of cases with characteristic of (30%)

P_D_ = Population proportion of control with characteristic of (21%)

Z = Constant, Standard normal deviance (1.96 for 95%)

Therefore, the sample size obtained was 90 pairs. Adding 13% attrition rate, the sample size obtained per group was 102, and this made a total sample enrolled for this study 204 women. Using a ratio of 1 case to 2 controls, there were a total of 68 cases and 136 controls in this study.

The stratified proportionate sampling method was initially used to obtain a representative sample from each hospital using the formula ni = (Ni/Nt)*n^16^where Ni = The average number of post-delivery mothers in each hospital for one week (Kivunge 138, Makunduchi 138 and Mnazi-mmoja hospital 352) Nt =The total number of post-delivery mothers in all the selected three hospitals for one week (628), n = The sample size for this study and ni = The number of a sample from each hospital which are (Kivunge= 45, Makunduchi =45, Mnazi Mmoja 114). Within the hospital, a group of cases and controls were identified, and simple random sampling by lottery with replacement method was used to select the required number of cases and the controls.

### Data Collection

An interviewer-administered questionnaire was used to collect data. Three nurse-midwives with advanced diploma levels were selected as research assistants. Prior to data collection, these assistants were trained on the data collection method and tool, as well as how to ask questions and complete the questionnaire. Each research assistant and the principal investigator were assigned to a single hospital at a time, and they collected data separately. The data collection was conducted at the post-delivery ward on a daily basis during the morning and evening hours whereby, immediately after delivery, the birth outcome was assessed to identify the cases and the controls, and then mothers in both groups were interviewed within the first 4 hours for their fears experienced during pregnancy, labour and delivery.

### Measurement of Level of Fear

The tool used to measure fear was the ‘Childbirth Attitude Questionnaire' which was adopted and modified from Dönmez et al. ^17^ Fear was measured using an index score, which was computed based on 19 items on a Likert scale with Strongly Disagree (SD=1), Disagree (D=2), Agree (A=3), and Strongly Agree (SA=4). Fear during pregnancy consisted of 7 items (fear of abortion, morning sickness, loss of appetite, change in body shape, thinking of date of delivery, change in partner behaviour, and fear of death). Fear during labour had 5 items (fear of pushing, fear of labour pain, fear of having baby with abnormalities, fear of heavy bleeding, and fear of death), while fear during delivery was measured using seven items (fear of pushing, fear of tears, fear of severe bleeding, fear of C/S, fear of the kind of baby that should have, fear of touching a baby, and fear of death). Strongly disagree and disagree were combined to form disagree while agree and strongly agree were combined to form agree. The fear index was derived using principal component analysis (PCA) in order to reduce a larger set of a variable into a smaller set if the variable is highly correlated and measuring the same underlying construct that does not sufficiently represent the construct of interest. During the PCA analysis, all items had a commonality of 0.3 and a factor loading of 0.5 hence were retained. The distribution of the fear index score was then divided into five categories (quintiles), each with approximately 20% of the population. The quintiles were named as the lowest, low, middle, high, and highest levels of fear.

### Data Analysis

Data was analysed using the Statistical Package for Social Sciences (SPSS) version 20.0. Descriptive statistics were used to describe the demographic characteristics of the respondents using percentage and frequency. A chi-square test was used to determine the relationship between fear experienced during pregnancy, labour and delivery, and birth outcomes. Inferential statistics using a logistic regression model were used to assess the association between fear during pregnancy, labour and delivery and birth outcomes. The odds ratio (OR) with its 95% CI was reported and the significance level was set at *p-value <.05*.

### Ethics Approval and Consent to Participate

The University of Dodoma (UDOM) Research Ethical Committee provided ethical clearance for this study with a reference number UDOM/DRP/134/VOL VII/36 and the ethical clearance for conducting research in Zanzibar was approved by the Zanzibar Medical Research Committee with reference number OMPR/M.95/C.6/2/VOL.XVII/92. A research permit was given by the Zanzibar Research Committee from the Office of Chief Government Statistician. The purpose of the study was explained to the participants and a written informed consent was obtained. Participants were allowed to withdraw from the study and be assured that obtained information was considered confidential and no harm associated with the study or means of data collection. Participants that were willing to participate were included. The participants that were found with health issues like diabetes and heart diseases were excluded. The undesirable outcome was managed according to the guideline and hospital protocol.

## RESULTS

### Characteristic of the Study Participants

A total of 204 post-delivery women participated in this study, which is equivalent to 100% of the response. Out of 204 participants, a majority 157(76.96%) were in the age group of 25 to 35 years which is the prominent age group among cases 51(75.0%) and among controls 106 (77. 94%). All participants were married and the majority of all cases 49(72.06%) and all control 92(67.65%) were less than para 4. Most participants in all cases 47(69.1%) and all controls 69(50.7%) completed secondary school, and slightly more than half of all cases 38(55.9%) and controls 74(54.4%) were unemployed. There was no statistical difference between cases and controls in all of the demographic characteristics (Table 1). The following birth outcomes were observed: Postpartum Hemorrhage (PPH) 14(21%), prolonged labour 12(18%), and ruptured uterus 1(1%). More details are shown in Table 2.

**TABLE 1:** Characteristics of the Study Participants (N=204)

Variable	Total (n=204) n(%)	Case (n=68) n(%)	Control (n=136) n(%)	Chi-square (P-value)
Age				0.228 (.8924)
<25	19 (9.31)	7 (10.29)	12 (8.82)	
25-35	157 (76.47)	51 (75.00)	106 (77.94)	
>35	28 (27.95)	10 (14.71)	18 (13.24)	
Para				1.628 (.6531)
<4	142 (69.85)	49 (72.06)	92 (67.65)	
4-6	54 (50.74)	15 (22.06)	39 (28.68)	
7-9	6 (6.62)	3 (4.41)	3 (2.21)	
10-12	3 (2.94)	1 (1.47)	2 (1.47)	
>12	0 (0.00)	0 (0.00)	0 (0.00)	
Education				8.732 (.0682)
Illiterate	29 (28.68)	10 (14.71)	19 (13.97)	
Primary	39 (34.55)	8 (11.76)	31 (22.79)	
Secondary	116 (59.93)	47 (69.12)	69 (50.74)	
High school	11 (9.56)	2 (2.94)	9 (6.62)	
College/University	9 (7.35)	1 (1.47)	8 (5.88)	
Job				2.237 (.5248)
Employed	20 (8.38)	5 (7.35)	15 (11.03)	
Not employed	112 (55.14)	38 (55.88)	74 (54.41)	
Peasant	24 (22.06)	6 (8.82)	18 (13.24)	
Small business	48 (49.26)	19 (27.94)	29 (21.32)	

**TABLE 2:** Frequency Distribution of Birth Outcomes Observed (N= 68)

Birth outcome	n(%)
Birth asphyxia	11 (16.1)
Cesarean section	8 (11.7)
Fetal distress	4 (5.8)
Instrumental delivery	2 (2.9)
Intrauterine fetal demise	1 (1.4)
Low birth weight	5 (7.3)
Postpartum hemorrhage	14 (20.5)
Premature delivery	9 (13.2)
Prolonged labour	12 (17.6)
Ruptured uterus	1 (1.4)
Pre/eclampsia	1 (1.4)
Normal birth outcomes	136 (66.6)

### Fear During Pregnancy, Labour and Delivery

On a Likert scale, respondents were asked what they feared during pregnancy, labour, and delivery. Strongly disagree and disagree were combined to form disagree, and agree and strongly agree were combined to form agree. The results showed that, during pregnancy, the majority of women 155(75.98%) feared for death, a little less than half 86(42.16%) feared for thinking of the date of delivery, and only 29(14.21%) feared for change of body shape. The majority of 172(84.31%) feared death during labor, 136(66.66%) feared pushing the baby, and only 41(20.10%) feared having a baby with an abnormality. And during delivery majority 165(80.88%) feared for death, 109(53.43%) feared for tears and 100(49.02%) feared for undergoing caesarean section (Table 3).

**TABLE 3:** Frequency Distribution of Constructs of Fear during Pregnancy, Labour and Delivery (N=204)

Variable	Strongly Disagree n(%)	Disagree n(%)	Agree n(%)	Strongly Agree n(%)
During Pregnancy				
Abortion	121 (59.31)	24 (11.76)	33 (16.18)	26 (12.75)
Morning sickness	98 (48.04)	23 (11.27)	67 (32.84)	16 (7.84)
Loss of appetite	99 (48.53)	22 (10.78)	65 (31.86)	18 (8.82)
Change of body shape	116 (56.86)	59 (28.92)	17 (8.33)	12 (5.88)
Date of delivery	85 (41.67)	33 (16.18)	42 (20.59)	44 (21.57)
Change of partner behaviour	119 (58.33)	40 (19.61)	22 (10.78)	23 (11.27)
Death	46 (22.55)	3 (1.47)	17 (8.33)	138 (67.65)
During labour				
Pushing	60 (29.41)	8 (3.92)	64 (31.37)	72 (35.29)
Labour pain	64 (31.37)	11 (5.39)	67 (32.84)	62 (30.39)
Abnormalities of baby	111 (54.41)	52 (25.49)	24 (11.76)	17 (8.33)
Heavy bleeding	81 (39.71)	44 (21.57)	46 (22.55)	33 (16.18)
Death	30 (14.71)	2 (0.98)	16 (7.84)	156 (76.47)
During delivery				
Pushing	56 (27.45)	21 (10.29)	69 (33.82)	58 (28.43)
Tear	60 (29.41)	35 (17.16)	53 (25.98)	56 (27.45)
Severe bleeding	95 (46.57)	42 (20.59)	46 (22.55)	21 (10.29)
Fear of C/S	75 (36.76)	29 (14.22)	56 (27.45)	44 (21.57)
Abnormalities of baby	110 (53.92)	69 (33.82)	9 (4.41)	16 (7.84)
Touching of the baby	113 (55.39)	32 (15.69)	43 (21.08)	16 (7.84)
Death	36 (17.65)	3 (1.47)	13 (6.37)	152 (74.51)

### Level of Fear During Pregnancy, Labour and Delivery among Cases and Control

**FIGURE 1:**
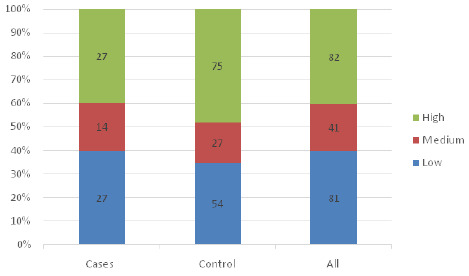
Level of Fear During Pregnancy Among Cases and Controls

**FIGURE 2:**
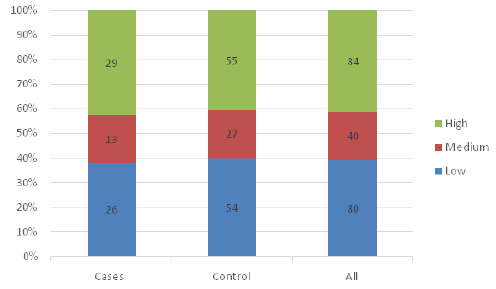
Level of Fear During Labour among Cases and Controls

**FIGURE 3:**
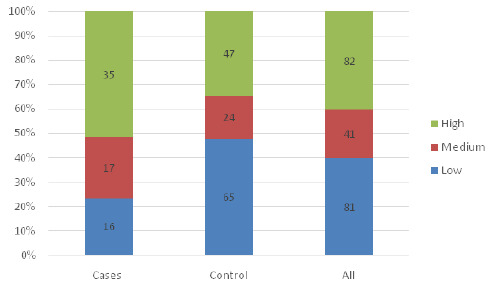
Level of Fear During Delivery among Cases and Controls

Figures 1, 2 and 3 show the distribution of fear levels among cases and control during pregnancy, labour, and delivery. The fifth quintiles were combined, lowest and low to form low, high and highest to form high and medium remained the same. The findings showed that during pregnancy, among cases, 27(39.7%) had a high level of fear during pregnancy compared to the controls, 75(40.4%). During labour, equal proportions of cases 29(42.6%) and of controls 55(42.4%) had a high level of fear, and during delivery, 35(51.4%) of cases had a high level of fear, while only 47(34.5%) of controls had a high level of fear. (Figure 1, 2 and 3).

The above-mentioned comparative graphical analysis of patterns of fear among cases and controls may provide a clear indication of the premise that there is association between them. To validate that premise, the chi-square test with a significance level of 5% was conducted, and the results showed that only fear during delivery was statistically significantly associated with having undesirable birth outcomes (χ^2^ =11.17, *p=.003*). The study showed no significant relationship between fear experienced during pregnancy (χ^2^ =0.02, *p=.9909*) and during labour (χ^2^ =0.09, *p=.9555*) with the birth outcome. Furthermore, the chi-square test was conducted to confirm if there are covariates other than fear that can significantly provoke undesirable birth outcomes, and the results showed none of the covariates (demographic variables) showed a significant association with birth outcomes (Table 4).

**TABLE 4:** Relationship between factor and birth outcome (N=204)

Variable	Level of fear		Chi-square	p-Value
	Case n(%)	Control n(%)		
Age			0.228	.8924
<25	7 (10.29)	12 (8.82)		
25-35	51 (75.00)	106 (77.94)		
>35	10 (14.71)	18 (13.24)		
Parity			0.4134	.5203
<4	49 (72.06)	92 (67.65)		
4+	19 (27.94)	44 (32.35)		
Job			2.237	.5248
Employed	5 (7.35)	15 (11.03)		
Not employed	38 (55.88)	74 (54.41)		
Peasant	6 (8.82)	18 (13.24)		
Small business	19 (27.94)	29 (21.32)		
Education level			3.6224	.1635
No formal education	10 (14.71)	19 (13.97)		
Primary	8 (11.76)	31 (22.79)		
Level of fear in Pregnancy			0.018	.9909
Low	27 (39.71)	54 (39.71)		
Medium	14 (20.59)	27 (19.85)		
High	27 (39.71)	55 (40.44)		
Fear during Labour			0.091	.9555
Low	26 (38.24)	54 (39.71)		
Medium	13 (19.12)	27 (19.85)		
High	29 (42.65)	55 (40.44)		
Fear during Delivery			11.167	.0038
Low	16 (23.53)	65 (47.79)		
Medium	17 (25.00)	24 (17.65)		
High	35 (51.47)	47 (34.56)		

Simple and multiple logistic regression analysis were then conducted to determine the sole effect of fear during delivery on birth outcomes. The results of the models showed that women who experienced a high level of fear during delivery were two times more likely to have undesirable birth outcomes than those who experienced a low level of fear (AOR = 1.941, *p=.051*) after adjusting for fear during labour and during pregnancy (Table 5).

**TABLE 5:** Logistic Regression Model for Association between Fear and Birth Outcome (N=204)

Variable	Un adjusted logistic model		Adjusted logistic model	
	OR(95%CI)	p-value	AOR(95%CI)	p-value
Fear in Pregnancy				
Low	Reference	1.000	Reference	
Medium/High	1.0 (0.55-1.81)			
Fear during Labour				
Low	Reference	.839	Reference	
Medium/High	1.0 (0.58-1.93)			
Fear during Delivery				
Low	Reference	.001	Reference	.051
Medium/High	2.9 (1.54-5.72)		1.94 (1.19-4.1)	

## DISCUSSION

Fear as one of the dimensions of pregnancy-related anxiety reported to negatively affect women's and infants' health. This study therefore determined the influence of fear during pregnancy, labour and delivery on birth outcomes in Unguja Zanzibar.

This study shows that all women experience some degree of fear at varying levels during pregnancy, labour, and delivery. The reason for having fear was not explored in this study. However, it has been observed in the qualitative study done in Mwanza –Tanzania that a lack of knowledge or understanding of what was normal was one among the reasons for many of the worries participants had about pregnancy.^4^ Fear of death was the most popular type of fear experienced during pregnancy, labour, and delivery. This was expected considering the high maternal mortality rates accompanied by a shortage of resources in this setting which may impair the quality of pregnancy-related services. ^18^

This study has found that only having fear during delivery is associated with having undesirable birth outcomes assessed in this study, which includes birth asphyxia, C/S, fetal distress, low birth weight, PPH, premature delivery, prolonged labour, ruptured uterus, and severe pre/eclampsia. Evidence shows that fear results in increases incortisol and norepinephrine, which are known to be associated with abnormal uterine contractions and subsequent obstetric complications.^19^ Despite the fact that this study did not specify the type of birth outcome related to fear experienced during pregnancy, labour, and or delivery, previous research has shown that fear during delivery is associated with elective C/S and emergency C/S. ^20^

This study, however, did not find a significant association between fear during pregnancy and labour with birth outcomes. However, evidence suggests that severely anxious mothers may feel overwhelmed and fatigued during pregnancy, which may impact their consistency of prenatal care, diet, and sleep habits, potentially contributing to poor birth outcomes.^13^ This study's findings are consistent with what has been reported in several studies that maternal fear or anxiety during pregnancy has effects on the length of labour,^21^ pre-eclampsia, prolonged labour and forceps delivery, ^22^ use of anaesthesia during delivery, ^23^ and preterm delivery.^24^Furthermore, literature shows that severe anxiety during pregnancy has a significant long term impact on newborns such as height, head circumference, and weight^25^ and an increased risk of giving birth to low birth weight babies and preterm births. ^26^ The effect of fear on newborn might be due to changes in the blood flow to the baby, making it difficult to carry oxygen and other important nutrients to the baby's developing organs.^27,28^

The inconsistent findings in this study can be due to lack of specificity. That is the specific dimensions of fear (eg, fear of pain, coping, or safety) may interact with specific birth outcomes (e.g., duration of labour or fetal heart rate deceleration). Therefore, the broad definition of fear and birth outcome in this study may be the reason for the inconsistent results. Moreover, the results of this study should be interpreted with caution as there are some limitations that are worth mentioning. The assumption used to calculate the sample size may have underestimated the sample, thus reducing the power of this study. Results are also limited by a failure to control for confounding variables, such as the kind of healthcare services received during pregnancy, labour, and delivery, which may have an influence on birth outcome and level of fear, or women's knowledge of the possibility of complications. Finally, the timing of the measurement of fear in this study may not be possible to distinguish whether the fear was present before delivery or whether the fear came as a result of adverse birth outcomes. The ideal design would have been to assess the presence of fear before labour or delivery and then briefly follow up the mothers to determine whether they developed adverse birth outcomes or not. Therefore, future studies should take these suggestions into consideration.

## CONCLUSION

This study established that, most women experience high level of fear during pregnancy, labour, and delivery. A high level of fear during delivery is associated with having undesirable birth outcomes but not during pregnancy and labour. The findings are of clinical importance as they highlight the need to integrate a universal screening intervention into the antenatal care services for early management.
